# Mechanism of the Effects of Storage Time and Matcha Addition on the Quality of Re-Steamed Bread: The Structure and Function of Gluten Protein and Starch

**DOI:** 10.3390/foods15132255

**Published:** 2026-06-23

**Authors:** Yi Liu, Qian Zhou, Lamei Li, Shan Gao, Yongrong Xiao, Yahui Zhang, Junfeng Cheng, Haihua Zhang

**Affiliations:** 1 College of Food and Health, Zhejiang Agriculture and Forestry University, Hangzhou 311300, China; liuyi@zafu.edu.cn (Y.L.);; 2 National Key Laboratory for Development and Utilization of Forest Food Resources, Zhejiang A&F University, Hangzhou 311300, China; 3 Shaoxing Economic Crop Technology Promotion Center, Shaoxing 312000, China; 4 Civil Aviation Transportation College, Sanya Aviation and Tourism Vocational College, Sanya 572000, China

**Keywords:** matcha, tea, gluten protein, starch, cold storage

## Abstract

This study aimed to investigate the effects of cold storage time and matcha addition on the multi-scale structure and functionality of gluten protein and starch in re-steamed bread. Results showed that prolonged cold storage destroyed the integrity of gluten networks by breaking disulfide bonds and altering protein secondary structures, accompanied by moisture loss and migration; meanwhile, starch retrogradation was significantly promoted, resulting in increased hardness and decreased specific volume. The addition of 0.5–1.0% of matcha stabilized disulfide bonds and inhibited starch retrogradation, thus alleviating quality decline. When the addition amount exceeded 1.0%, high concentrations of polyphenols depolymerized gluten proteins and accelerated moisture transfer, causing a further drop in specific volume. Pearson correlation analysis verified the close correlations between macroscopic quality and microstructural characteristics. This study explored the mechanisms underlying the effects of cold storage time and matcha addition on the quality of re-steamed bread, providing a systematic scientific basis for the application of tea flour products in cold storage.

## 1. Introduction

As society has advanced, traditional staple foods such as steamed bread have undergone gradual innovations. Currently, the application of cold storage technology has made re-steamed bread an indispensable part of the industrialization of staple foods. However, upon re-steaming, re-steamed bread has been observed to suffer from quality deterioration, including reductions in volume, increases in hardness, and moisture loss [1,2,3]. Some studies have revealed that cold storage causes protein denaturation, leading to the disruption of ionic and hydrophobic bonding forces, which in turn impairs the ability of the gluten network to retain water [4,5]. Cold storage also induces depolymerization of gluten proteins, resulting in irregular structures and compromised integrity [6]. Additionally, cold storage promotes the reorganization of starch structures, leading to starch retrogradation and subsequent quality degradation of the re-steamed bread [7,8]. However, existing research primarily focuses on individual components, with a lack of systematic analysis on the synergistic effects of multiple components and the regulatory effects of exogenous additives, leading to a lack of theoretical depth in the development of antifreeze strategies.

Tea, as a natural and excellent food additive, imparts a vibrant color and a subtle tea aroma to re-steamed bread. Additionally, tea polyphenols have been proven to possess significant antioxidant properties, effectively neutralizing free radicals in the body, thereby reducing oxidative damage and protecting cells from aging and disease. This characteristic has garnered widespread attention in health and beauty applications, making tea polyphenols a crucial component in many food additives. Numerous studies have focused on the interactions between polyphenols in tea-containing wheat flour products and wheat flour components, such as gluten proteins and starch granules [9,10]. The impact of tea polyphenols and their monomer (catechins) on gluten network strength had yielded inconsistent results. Some studies [10] have suggested that tea polyphenols and catechins enhance gluten network strength, leading to a more continuous and ordered structure in polyphenol-enriched noodles, while others [11] have reached contrary conclusions. These discrepancies may be attributed to variations in raw material types and addition amounts. Although the introduction of polyphenols can stabilize the gluten protein conformation through hydrogen bonding and hydrophobic interactions, and delay starch retrogradation [12,13,14], excessive addition may weaken the water-storage ability of the gluten protein, thus exacerbating cold storage damage [15]. The concentration-dependent regulatory mechanism remains unclear.

Therefore, to thoroughly investigate the effects of cold storage time and matcha addition on re-steamed bread quality, this study focused on chilled fresh re-steamed bread, the dominant commercial staple food in China that is conventionally stored and distributed at 0–4 °C, rather than frozen re-steamed bread preserved at −18 °C. Accordingly, 0 °C was adopted as the storage temperature to simulate actual industrial logistics conditions. The study explored the impacts of cold storage durations (0, 1, 3, 6, and 9 days) and matcha contents (0%, 0.5%, 1.0%, and 1.5% by flour weight) on the textural properties, moisture distribution, secondary structure of gluten proteins, chemical bonds, and starch ordering in re-steamed bread samples. It was hypothesized that matcha exerts concentration-dependent effects on re-steamed bread quality during cold storage. The study aimed to delve into the reasons behind quality deterioration due to cold storage and the effects of matcha addition on the structure and functionality of re-steamed bread, providing a systematic scientific basis for the application of tea-containing products in cold storage.

## 2. Materials and Methods

### 2.1. Materials

Wheat flour (13.7% protein content, 12.7% moisture content, *w*/*w*) purchased from Qinhuangdao Goldensea Foodstuff Industries Co., Ltd. (Qinhuangdao, China). Sugar purchased from Baolong Supermarket in Hangzhou, China. Freshly picked tea leaves (Longjing 43) obtained from a tea garden in Song Yang. Yeast purchased from Lesaffre (Mingguang) Co., Ltd. (Mingguang, China). Ellman reagent, 2-Amino-2-(hydroxymethyl)-1,3-propanediol (Tris), trichloroacetic acid (TCA) and all other reagents purchased from Shanghai Macklin Biochemical Co., Ltd. (Shanghai, China).

### 2.2. Preparation of Samples

Matcha (the content of fiber: 11.61%, protein: 31.12%, phenols: 15.28%, flavone: 8.39%): The freshly picked tea leaves were washed and steamed for 10 min. Then, the tea leaves were quickly pulverized into a powder within liquid nitrogen. The sample was passed through a sieve with a mesh size of 20 µm, and the sample passing through the sieve was subjected to vacuum freeze-drying treatment to obtain the final sample.

Analytical methods for matcha components: The dietary fiber was detected using the method of Huang [16], protein was detected using the method of Koláčková [17], total phenols were detected using the method of Yu [10], and total flavonoids were detected using the method of Shu [18].

A solution was prepared by combining 2 g of active dry yeast and 10 g of sugar with 100 mL of deionized water. The resulting mixture was blended with 200 g of wheat flour using a mixer (B20, Yuanmai Co., Ltd., Shanghai, China) at low speed for 3 min. Subsequently, varying concentrations of 0%, 0.5%, 1%, and 1.5% matcha (based on the weight of the wheat flour, *w*/*w*) was added to the mixture, which was then subjected to sequential mixing phases: medium speed for 8 min and finally at low speed for 3 min. The prepared dough was left at room temperature for 10 min. It was then divided into 50 g portions, shaped into balls, and stayed for 30 min at 35 °C with 80% relative humidity. The dough was steamed for 15 min in a steamer (KZ-60D, Yuanmai Co., Ltd., China). After steaming, all samples were cooled to 25 °C. The 0-day group was immediately re-steamed for analysis without cold storage. The remaining samples were stored in polypropylene bags at 0 °C for 1, 3, 6, and 9 days, then re-steamed and cooled to 25 °C before further analysis. Three independent dough preparations were conducted for each treatment. Each dough preparation produced 6 uniform re-steamed bread (50 g each). For each test, 3 re-steamed breads were randomly selected from each replicate. All measurements were performed on the central part of the re-steamed bread.

### 2.3. Specific Volume Analysis

Initially, millet was used to fill a 500 mL beaker, which was then emptied for later use. After re-steaming, the cooled re-steamed bread was put into the beaker. Then, it was refilled with millet to its original volume. The volume of millet remaining was used to determine the volume of the re-steamed bread. The specific volume of the re-steamed bread was calculated as the volume divided by their weight [3].

### 2.4. Texture Properties Analysis

The re-steamed bread samples were sliced into uniform 15.0 mm thick pieces, with two slices selected from the center of each sample. A texture analyzer (iTexture, Zhe Ke Co., Ltd., Hangzhou, China) was employed to evaluate textural properties through Texture Profile Analysis (TPA) [19]. Measurements were conducted using a P/36R probe under the TPA parameters: test speed of 2.0 mm/s, compression ratio of 45%, dwell time of 3 s, trigger force of 15 g, and data acquisition rate of 400 pps.

### 2.5. Total Water Content Analysis

The total water content of the re-steamed bread was measured following the method described by Duan [7,8]. Initially, the weight of the empty dried plates was recorded. Subsequently, 3 g of re-steamed bread was placed on the plate, which was then placed in an electric thermostatic blast dryer (DHG-9070, Yiheng Co., Ltd., Shanghai, China) at 105 °C stay for 6 h. After drying, the plate was removed from the dryer and allowed to cool to 25 °C in a desiccator. This process was repeated three times for each measurement to ensure accuracy. The total water content (*w*/*w*) of the re-steamed bread was calculated using Equation (1):

(1) Total water content (%) = (*W*_1_ − *W*_2_)/*W*_1_ × 100

where W_1_ is weight of re-steamed bread, g; W_2_ is weight of dried re-steamed bread, g.

### 2.6. Low Field Nuclear Magnetic Resonance (LF-NMR) Analysis

The changes in moisture distribution of re-steamed bread during the cold storage process were analyzed using a low-field nuclear magnetic resonance imager (MesoMR23-060V-I, Niumag Co. Ltd., Suzhou, China) [20]. Before measurement, 2 g of re-steamed bread crumbs were cut into 5 mm cubes and placed into a 20 mm radius nuclear magnetic resonance special glass tube, and the parameters were set as follows: sample frequency = 100 kHz, accumulation times = 2, number of echoes = 1000, time of echoes = 1.00 ms. Measurements were performed using the Carr–Purcell–Meiboom–Gill (CPMG) pulse sequence parameters. The inversion algorithm (CONTIN) was used to invert the acquired data to obtain the spectra, the spin–spin relaxation time (T2) values, and the corresponding proton signal amplitudes calculated for each sample.

### 2.7. The Free Sulfhydryl Content of Samples

A modified version of the method described by Zhao [5] was employed. Sample powder containing 50 mg of protein was added to 5.0 mL of Tris-Gly-8 mol/L Urea buffer solution. After vortexing for 3 min, the mixture was centrifuged at 8000 r/min for 10 min. 2 mL supernatant was taken and diluted by a factor of five. Under light-protected conditions, 200 µL of 10 mol/L Ellman reagent was added to the collected supernatant and reacted for 20 min. Absorbance at 412 nm was measured within 30 min using a microplate reader (HBS-1096A, De Tie Co., Ltd., Nanjing, China). The content of sulfhydryl groups (SH) was calculated using the formula:

(2) SH (µmol/g) = 73.53 × *A*_412_ × *D*/*C*

where *A*_412_ is the absorbance of the sample at 412 nm; *C* is the sample concentration in mg/mL; and *D* is the sample dilution factor.

### 2.8. The Disulfide Bonds of Samples

A modified version of the method described by Zhao [5] was employed. Mix 0.1 mL of the supernatant obtained from the above centrifugation with 10 µL of β-mercaptoethanol, 400 µL of the buffer solution, and 1 mL of 12% trichloroacetic acid (TAC) solution. The mixture was incubated at 25 °C for 2 h. The sample was centrifuged at 8000 r/min for 10 min, and the precipitate was cleaned three times with 500 µL of 12% TCA solution before being dissolved in 1 mL of the buffer solution. 10 µL of the Ellman reagent was added to the precipitate, and the reaction was allowed to proceed for 20 min. The absorbance of samples at 412 nm was measured using the microplate reader. The disulfide bond content was calculated as follows:

(3) Disulfide bond content (µmol/g) = (Total thiol content − Free thiol content)/2

### 2.9. Fourier Transform Infrared (FTIR) Spectrometer Analysis

The freeze-dried sample powder (1.0 mg) was combined with 100 mg of KBr, and the resulting mixture was compressed into a sheet. The 4000 to 400 cm^−1^ spectra were collected using FTIR (Nicolet iS20, Thermo Fisher Scientific Inc., Waltham, MA, USA) [5]. The resolution was set at 4 cm^−1^ and 32 scans were carried out.

The baseline correction of amide I band (1600–1700 cm^−1^) and disulfide bond configurations (490–550 cm^−1^) was performed with both OMNIC 8.2 software. Further spectral analysis was performed using Peakfit v4.12 software, involving peak deconvolution and second derivative analysis. The goodness of fit for peak fitting was set at (R^2^ > 0.99). The spectra in the 1200–800 cm^−1^ range were deconvoluted using OMNIC 8.2 software, with a resolution enhancement factor and width set to 2.1 and 38 cm^−1^, respectively. The peak intensities at 1045 cm^−1^ and 1022 cm^−1^ were calculated.

### 2.10. X-Ray Diffractometer (XRD) Analysis

The freeze-dried re-steamed bread powder was placed in the sample holder and subjected to X-ray diffraction analysis using a D2 Phaser X-ray diffractometer (Bruker, Corporation, Karlsruhe, Germany), following the method of Lv [12]. All samples were ground and sieved through a 200-mesh sieve. The diffractometer was operated at a scanning rate of 2°/min and a step size of 0.02°. The amorphous background was separated using Jade 6.0 software by manually defining baseline points at 2θ = 5°, 12°, 25°, and 35°, then fitting the background with a cubic spline function. The relative crystallinity was calculated as the ratio of the area of crystalline peaks to the total area after background subtraction.

### 2.11. Statistical Analysis

This study adopted a 5 × 4 full factorial design with 5 cold storage times (0, 1, 3, 6, 9 days) and 4 matcha addition levels (0%, 0.5%, 1.0%, 1.5%). Independent experiments were repeated at least three times, and the experimental data were presented in the form of mean ± standard deviation. All measured parameters showed significant interaction effects (*p* < 0.05) between storage time and matcha addition. Duncan’s multiple range test was used for post hoc comparisons at *p* < 0.05. The analysis was performed using the SPSS 27.0 Statistical Software Program. Heatmap analysis was conducted using Origin 2025, based on the Pearson correlation coefficient.

## 3. Results

### 3.1. Specific Volume of Re-Steamed Bread Samples

**Figure 1:**
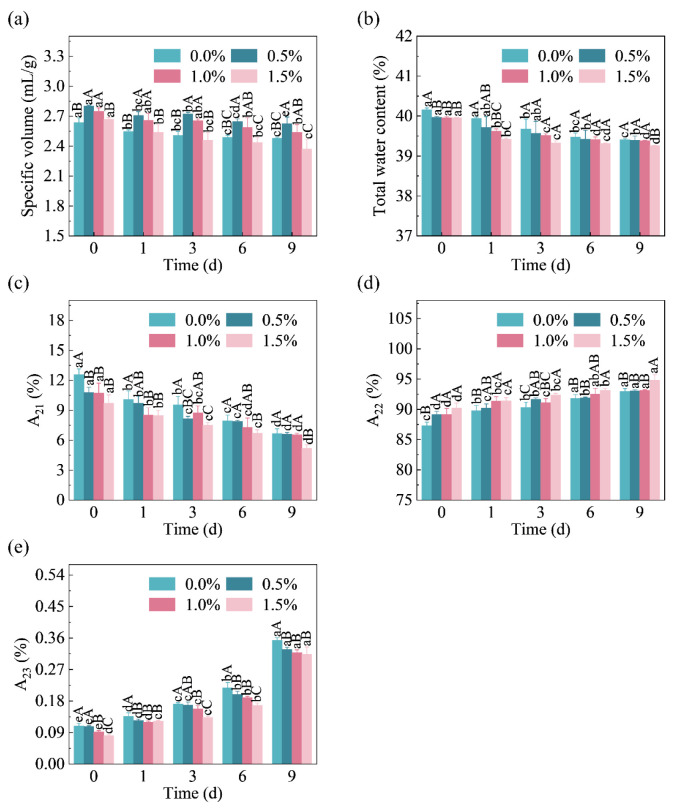
Changes in specific volume (**a**), total water content (**b**) and water distribution (**c**–**e**) in re-steamed bread with different matcha addition amounts. ((**c**–**e**) A21—area of bound water, A22—area of semi-bound water, A23—area of free water.) Letters represent a significant difference between the data in the same column (*p* < 0.05).

The specific volume is an indicator of the expansion and gas retention capacity of re-steamed bread [20]. The effect of cold storage on the specific volume of re-steamed bread samples was illustrated in Figure 1a. At the same content of matcha, the specific volume of the re-steamed bread samples decreased with increasing storage time. This reduction was attributed to the increase in the amount of freezable water during the cold storage process, which led to changes in moisture state and damage to the gluten network structure [21]. At the same storage time, the specific volume of the re-steamed bread initially increased and then decreased as the content of matcha increased. Compared to the control group without matcha, the specific volume of the sample with 0.5% matcha increased by 6.06% at day 0 of storage, while the specific volume only increased by 1.14% when the matcha concentration was raised to 1.5%. A small amount of matcha resulted in an increase in specific volume, which was due to the polyphenols enhancing the gluten network structure through hydrogen bonding [22]. The increase in specific volume with a small amount of matcha is consistent with conclusions drawn from studies on bread made with green matcha [22]. However, at day 9 of storage, the specific volume of the re-steamed bread with 1.5% matcha decreased by 9.54% compared to those with 0.5% matcha. The addition of polyphenolic substances leads to hydrophobic and hydrogen bonding interactions between the ingredients and polyphenols, resulting in changes in the secondary conformation of proteins, which in turn affects the specific volume [11]. Therefore, changes in molecular conformation and gluten proteins during the cold storage of matcha re-steamed bread account for the variations observed in their specific volume. Therefore, the difference in specific volume of re-steamed bread raised from the synergistic effect of structural deterioration caused by refrigeration and alterations in dough fermentation characteristics. CO_2_ produced by yeast fermentation is the basis for the formation of the porous structure [23]. High concentrations of tea polyphenols can inhibit yeast glycolytic metabolism, reduce gas production capacity, and lead to insufficient dough expansion. Meanwhile, the interaction between tea polyphenols and gluten proteins changes the rheological properties of the samples and reduces its gas-holding capacity [24]. The initial structure formed during fermentation has an additive effect with refrigeration-induced deterioration, making samples with poorer structural stability more prone to shrinkage and collapse during refrigeration, ultimately resulting in a significant decrease in specific volume (*p* < 0.05).

### 3.2. The Texture Properties of Re-Steamed Bread Samples

As shown in Table 1, the effects of matcha content and cold storage duration on five texture parameters of re-steamed bread—hardness, springiness, cohesiveness, adhesiveness, and chewiness—were assessed. With the increase in cold storage time, the hardness and chewiness of all samples significantly increased (*p* < 0.05), while elasticity, adhesiveness, and cohesiveness gradually decreased (*p* < 0.05). At 9 days of storage, the hardness of the control samples increased by 52%, and chewiness increased by 88%, whereas springiness, adhesiveness, and cohesiveness decreased by 25%, 18%, and 18%, respectively. This indicated that cold storage negatively affected the texture quality of steamed bread, which is consistent with several studies [25]. This effect was attributed to the growth of ice crystals disrupting the gluten network [25,26], or it could be due to moisture loss affecting the formation or breakage of hydrogen bonds between starch molecules or proteins and starch [11].

Before 6 days of storage, the addition of matcha had no significant effect on hardness (*p* > 0.05). However, at 9 days of storage, increasing the matcha content from 0.5% to 1.5% resulted in a 30.54% increase in hardness, indicating that a higher matcha content exacerbated the decline in texture quality during long-term cold storage. This is because moisture loss is not significant with short-term cold storage, thus its impact on texture characteristics is less pronounced. In contrast, during prolonged cold storage, excessive moisture loss leads to competition for water absorption by polyphenols, fibers, and polysaccharides in matcha [15], which becomes more evident in texture properties.

### 3.3. The Moisture Distribution of Re-Steamed Bread Samples

After cold storage, the moisture state of the re-steamed bread was characterized through LF-NMR by measuring the spin–spin relaxation time. T21 (0.01~3.05 ms), T22 (3.05~75 ms), and T23 (75~500 ms) represented tightly bound water, semi-bound water, and free water, respectively [27]. The areas under the T21, T22, and T23 curves were calculated and named as A21, A22, and A23 (Figure 1c–e).

In all fresh re-steamed bread (0 days), it was found that the moisture primarily existed as bound water (94% of total moisture) and immobilized water (5%), while free water accounted for less than 0.5%. This was attributed to the formation of an ordered gel network after the starch granules absorbed water and swelled. This structure, through physical hindering and chemical bonding, inhibited the migration of free water, promoting its conversion into bound water and semi-bound water [28]. Under the same matcha content, as the cold storage time increased, the total moisture content (Figure 1b) and the content of tightly bound water decreased, while the contents of semi-bound water and free water increased. This indicated that with prolonged cold storage, the re-steamed bread progressively lost moisture, and the tightly bound water transitioned into semi-bound and free water. Cold storage altered the moisture state in the re-steamed bread, increasing the mobility of the water. Cold storage caused some of the tightly bound water, associated with gluten proteins or starch, to convert into semi-bound and free water, leading to greater water loss during re-steaming. Additionally, during cold storage, the gluten network was disrupted, and microstructural cracks appeared, resulting in a reduced water-holding capacity of the proteins [29].

Under the same storage time, as the content of matcha increased, the total moisture content decreased, with a downward trend observed in both tightly bound water and free water, while semi-bound water exhibited an increasing trend. This was attributed to the competition between the hydroxyl groups in matcha and the hydroxyl groups in water for binding sites on the gluten proteins, leading to increased water mobility [30,31].

### 3.4. The Content of Free Sulfhydryl and Disulfide Bonds of Re-Steamed Bread Samples

**Figure 2:**
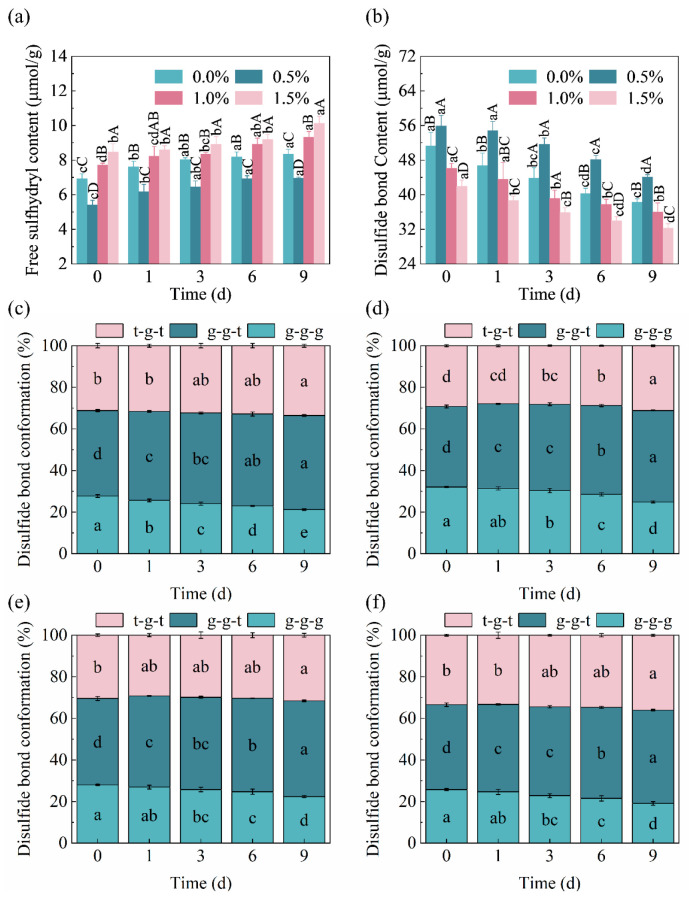
Changes in the contents of free sulfhydryl (**a**), disulfide bonds (**b**) and their conformations (**c**–**f**) in re-steamed bread with different matcha addition amounts. ((**c**–**f**) represented matcha additions of 0%, 0.5%, 1%, and 1.5%.) Letters represent a significant difference between the data in the same column (*p* < 0.05).

The changes in the content of free sulfhydryl in the re-steamed bread samples were illustrated in Figure 2a, while the changes in disulfide bond content were shown in Figure 2b. The variations in the content of free sulfhydryl are generally correlated with the changes in the content of disulfide bonds, both of which play crucial roles in protein aggregation and the maintenance of a stable gluten network structure [29]. Under the same matcha addition, all samples exhibited an increase in the content of free sulfhydryl with extended cold storage time, while the content of disulfide bonds displayed an opposite trend. This indicated that cold storage caused a redistribution of moisture within the re-steamed bread samples and the formation of ice crystals, which led to the rupture of intermolecular disulfide bonds and the depolymerization of proteins, resulting in an increase in the content of free sulfhydryl [6].

At the same cold storage time, as the matcha addition increased from 0.5% to 1.5%, the content of free sulfhydryl increased, while the content of disulfide bonds decreased. Specifically, when the matcha content was 0.5%, the content of free sulfhydryl was significantly lower than that of other sample groups (*p* < 0.05), and the content of disulfide bonds was significantly higher than in other groups (*p* < 0.05). This was attributed to the reducing properties of polyphenols in matcha, which disrupted the disulfide bonds in gluten proteins and led to an increase in the content of free sulfhydryl, thereby affecting the formation of the gluten network structure in re-steamed bread samples [10,32]. However, with a small amount of matcha (0.5%) added, the interaction between the polyphenols and gluten proteins promoted the formation of the gluten network, resulting in a significant increase in disulfide bond content and a significant decrease in the content of free sulfhydryl (*p* < 0.05). Conversely, excessive addition of matcha (≥1.0%) led to an accumulation of phenolic compounds, which caused exchanges of sulfhydryl or disulfide bonds within the gluten network and the breaking of hydrogen bonds [9]. This ultimately resulted in the depolymerization of gluten into smaller molecular proteins and the deterioration of the gluten network [33].

### 3.5. The Disulfide Bond Conformation of Re-Steamed Bread Samples

Disulfide bonds, which can link different peptide chains or different regions of the same peptide chain, serve as the primary force in maintaining the three-dimensional network structure of gluten proteins. Peaks within the 545–505 cm^−1^ range in the infrared spectra represent the disulfide bond conformations in the re-steamed bread samples. Specifically, the 506–515 cm^−1^ range corresponds to gauche-gauche-gauche (g-g-g), the 515–525 cm^−1^ range to gauche-gauche-trans (g-g-t), and the 525–545 cm^−1^ range to trans-gauche-trans (t-g-t) [34] (Figure 2c–e).

Under identical matcha addition levels, the content of relatively stable g-g-g conformation in the cold re-steamed bread decreased with prolonged cold storage time, while the content of unstable g-g-t and t-g-t conformations increased. When matcha was added at 1.5%, after 0 to 9 days of cold storage, the g-g-g content decreased by 25.79% (*p* < 0.05), the g-g-t content increased by 9.93% (*p* < 0.05), and the t-g-t content increased by 7.71% (*p* < 0.05). This indicated that cold storage reduced the stability of disulfide bonds in gluten [21,35]. For the same cold storage time, as the matcha concentration increased from 0.5% to 1.5%, the g-g-g conformation contents decreased, while the g-g-t and t-g-t conformation contents increased. The results, in conjunction with the disulfide bond content data, suggested that increased matcha addition led to a progressive degradation of the gluten protein structure and a decrease in disulfide bond content, resulting in less stable disulfide bond conformations. For a small amount of matcha (0.5%), the results indicated a higher content of intramolecular disulfide bonds (primarily g-g-g conformation) in the cold re-steamed bread, suggesting that a small amount of matcha could enhance disulfide bond stability. This finding was consistent with the results for free thiol and disulfide bonds in the gluten protein, and corroborated the results for the specific volume and texture of the re-steamed bread.

### 3.6. The Secondary Structure of Re-Steamed Bread Samples

**Figure 3:**
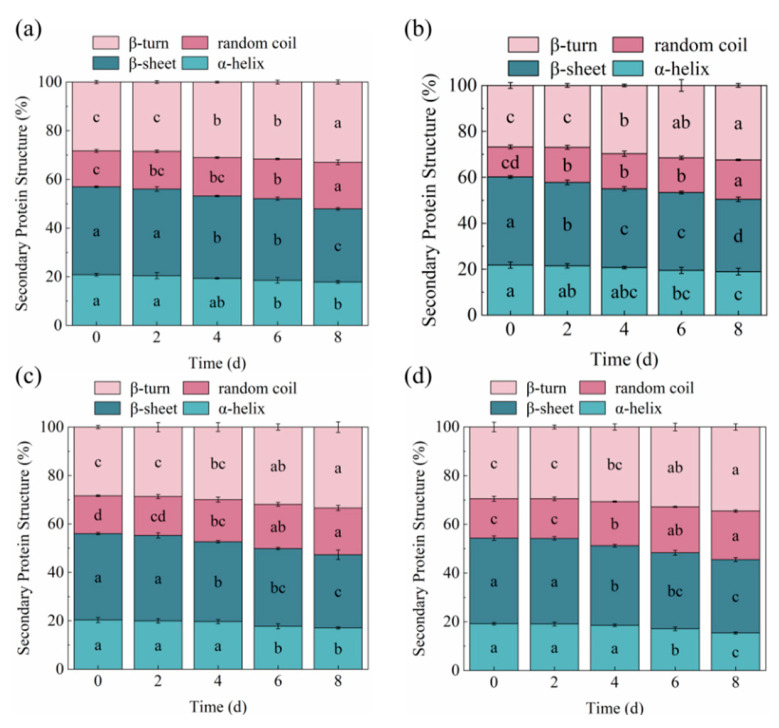
Changes in the content of the secondary structure of gluten protein in re-steamed bread with different matcha addition amounts. ((**a**–**d**) represented matcha additions of 0%, 0.5%, 1%, and 1.5%.) Letters represent a significant difference between the data in the same column (*p* < 0.05).

FTIR spectra were employed to determine the secondary structure of proteins. Peaks in the 1600–1640 cm^−1^, 1640–1650 cm^−1^, 1650–1660 cm^−1^, and 1660–1700 cm^−1^ ranges corresponded to β-sheet, random coil, α-helix, and β-turn structures, respectively [11], as shown in Figure 3. The content of secondary structure was closely related to protein stability, with α-helix and β-sheet structures being relatively stable and orderly, where α-helix is maintained by intra-chain hydrogen bonds [4].

Under identical matcha addition levels, the content of β-turn and random coil in the re-steamed bread samples increased with prolonged cold storage, while the content of α-helix and β-sheet decreased. This indicated a partial conversion of α-helix to β-turn during cold storage, consistent with Wang’s finding [4]. This change was due to ice crystals disrupting the hydrogen bonds that maintain gluten protein secondary structure, exposing hydrophilic and hydrophobic regions and leading to protein denaturation [27]. After 9 days of cold storage, the total content of the ordered gluten protein structure (α-helix + β-sheet) in the 0% matcha group decreased by 16.22% (*p* < 0.05). This indicated that cold storage reduced the stability of the secondary structure of gluten proteins. Furthermore, cold storage resulted in an increase in fluidity of moisture, suggesting a reduction in the ability of gluten proteins to bind with water, which led to the rearrangement of intermolecular/intramolecular hydrogen bonds in the protein network [5,10]. With the content of matcha increasing from 0.5% to 1.5% under the same storage time, the gluten protein secondary structure shifted towards an unstable state. The formation of β-sheet structures was facilitated by disulfide bonds between gluten proteins [33]. This result and the results of the disulfide bond content and conformation indicated that matcha addition disrupted disulfide bond, leading to a reduction in β-sheet structures and consequently causing gluten protein expansion and dissociation. The diffusion of polyphenol-rich matcha into the gluten network resulted in extensive hydrogen bond breakage and disulfide bond disruption, causing a transition to a less stable secondary structure and damaging the gluten structure [10,32]. Additionally, fibers in matcha hindered the formation of disulfide bonds with gluten proteins [9], impacting the quality of the re-steamed bread. However, the proportion of α-helix to β-sheet structures increased with 0.5% matcha addition compared to the structural composition of the other matcha groups. It was suggested that there is a protective effect on gluten proteins of a small addition of matcha, as corroborated by the results of disulfide bond content, disulfide bond conformation, and texture properties.

### 3.7. Long-Range Order of Starch in Re-Steamed Bread Samples

**Figure 4:**
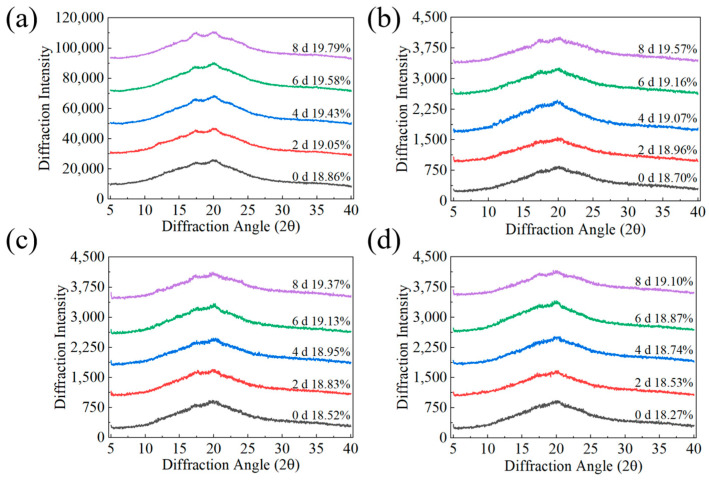
X-ray diffraction patterns of re-steamed bread with different matcha addition amounts. (**a**–**d**) represented matcha additions of 0%, 0.5%, 1%, and 1.5%.

The changes in the relative crystallinity of starch in re-steamed bread samples were illustrated in Figure 4. From the figure, it can be observed that the un-cold samples (0 day) exhibited a single peak at 20° (2θ), which corresponds to the V-type crystalline peak attributed to the formation of amylose-lipid complexes [36]. However, when the cold storage duration exceeded one day, distinct double peaks were observed at 17° and 20° (2θ). This indicated that starch retrogradation occurred during cold storage, primarily due to the reorganization and alignment of the short side chains of amylopectin, leading to the formation of B-type crystalline polymers.

Under the same matcha addition condition, the crystallinity increased (*p* < 0.05) with prolonged cold storage. This suggested that starch retrogradation took place during the cold storage process, accompanied by an increase in the ordered structure of the starch. Retrogradation led to the formation of a three-dimensional network structure, which increased the hardness of starch-containing foods, consistent with the results of texture and specific volume analyses. The retrogradation of amylose was found to determine the initial hardness of starch gels and the viscosity of processed foods. Long-term changes in the gel structure and the crystallinity of processed starch, including the aging of re-steamed bread, were considered to be related to the retrogradation of amylopectin [37]. Amylose retrogradation is a rapid process that completes within 24 h after cooling, contributing to the initial firming of fresh bread, while amylopectin retrogradation is a slow, continuous process that occurs over days to weeks, driving the long-term staling phenomenon observed in this study. This two-stage retrogradation kinetics precisely explains why the hardness of our re-steamed bread samples increased sharply in the first 3 days of cold storage and then continued to rise at a slower rate thereafter [38,39,40,41]. At same cold storage duration, an increase in matcha addition led to a decrease in relative crystallinity (*p* < 0.05), suggesting that matcha addition inhibited starch retrogradation. Some studies have observed similar results with the addition of tea polyphenols to starch, demonstrating that polyphenols could inhibit starch retrogradation [13]. This effect was attributed to the interaction between the phenolic hydroxyl groups in matcha and the hydroxyl groups on the starch chains, forming complexes that subsequently inhibited starch retrogradation [32].

### 3.8. Short-Range Order of Starch in Re-Steamed Bread Samples

The short-range ordered molecular structure of the double helices in starch molecules can be determined using Fourier transform infrared (FTIR) spectroscopy. The peak intensities at 1045 cm^−1^ and 1022 cm^−1^ are associated with the ordered structure of starch and the orientation of hydrogen bonds between CH and CH_2_ groups within the CH_2_OH moieties. The value of 1045/1022 cm^−1^ reflects the degree of order in the starch; an increase in this value during retrogradation corresponds to an enhancement in structural order.

As shown in Table 1, under the same levels of matcha addition, the value of 1045/1022 cm^−1^ of all samples increased with prolonged cold storage. This indicated that cold storage led to starch retrogradation and an increase in order, consistent with the XRD results. Conversely, under the same cold storage duration, an increase in matcha addition resulted in a decrease in the value of 1045/1022 cm^−1^. Specifically, when un-cold, the value of 1045/1022 cm^−1^ of samples with 0.5% matcha decreased by 2.74% compared to the control (*p* > 0.05), while samples with 1.5% matcha showed a 7.53% reduction (*p* < 0.05). This suggested that the addition of matcha delayed starch retrogradation. The phenolic compounds abundant in matcha are known to engage in non-covalent interactions with starch, including hydrogen bonding and hydrophobic, electrostatic, and ionic interactions [42]. These interactions hinder the formation of the double helical structure of amylopectin, thus interfering with starch retrogradation [43].

### 3.9. Correlation Analysis

**Figure 5:**
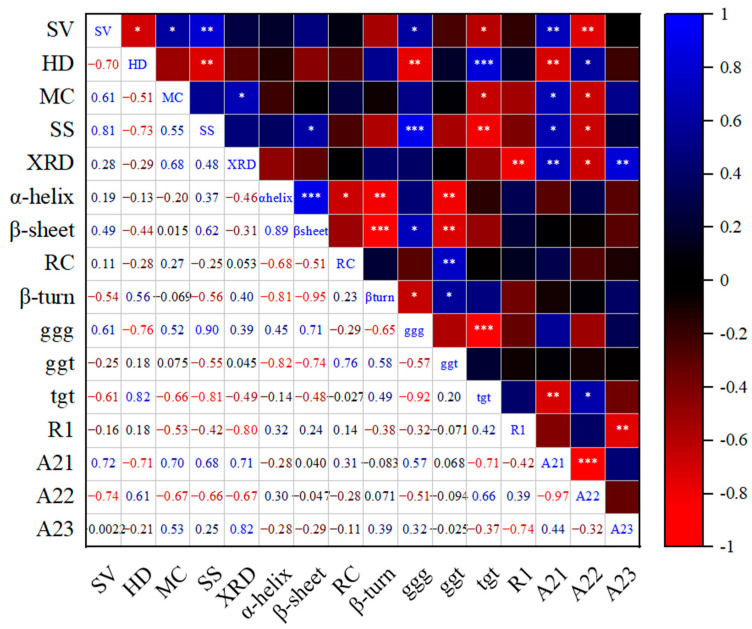
Correlation analysis of each index of re-steamed bread. SV, specific volume of re-steamed bread; HD, hardness of re-steamed bread; MC, total moisture content of re-steamed bread; SS, disulfide bond content of gluten protein in re-steamed bread; XRD, relative crystallinity of starch in re-steamed bread; α-helix, α-helix content of gluten protein in re-steamed bread; β-sheet, β-sheet content of gluten protein in re-steamed bread; RC, random coil content of gluten protein in re-steamed bread; β-turn, β-turn content of gluten protein in re-steamed bread; ggg, g-g-g conformation content of disulfide bonds in re-steamed bread; ggt, g-g-t conformation content of disulfide bonds in re-steamed bread; tgt, t-g-t conformation content of disulfide bonds in re-steamed bread; R1, ratio of starch absorption peaks at 1045 cm^−1^ and 1022 cm^−1^ in re-steamed bread; A21–A23, peak area ratio of T21, T22 and T23 in re-steamed bread. *, *p* ≤ 0.05, **, *p* ≤ 0.01, ***, *p* ≤ 0.001.

Pearson correlation analysis was used to explore the correlation between the macroscopic quality and multiscale structure of re-steamed bread samples under the combined effects of cold storage and matcha addition (Figure 5). The results showed no significant correlation between starch crystallinity (XRD) and hardness (HD). Polyphenols and dietary fiber in matcha can inhibit starch recrystallization through steric hindrance [44,45]; meanwhile, polyphenols can suppress α-amylase activity and alter the degree of starch hydrolysis [46,47,48], thereby weakening the effect of starch retrogradation. In addition, changes in the gluten network structure also affect hardness. Hardness was significantly negatively correlated with A21 (r = −0.71, *p* < 0.05) and significantly positively correlated with A22 (r = 0.61, *p* < 0.05). Cold storage promoted the conversion of strongly bound water to immobile water, exacerbating sample hardening, while the hydrophilic components in matcha retarded water migration and improved the storage quality of samples.

There is a significant correlation between disulfide bond conformation and the specific volume (SV) of samples. Specific volume (SV) is significantly negatively correlated with hardness (HD) (r = −0.70, *p* < 0.05), and both are significantly correlated with g-g-g and t-g-t conformations. An appropriate flexible structure ensures good extensibility and gas-holding capacity of the gluten network. Abnormal protein conformation leads to network disruption or excessive crosslinking, resulting in decreased specific volume and increased hardness [15]. On one hand, polyphenols regulate yeast metabolism and carbon dioxide production [49]. On the other hand, they alter the structure of gluten proteins through non-covalent interactions [30], affecting the specific volume of samples from both fermentation performance and gluten network structure. The quality of re-steamed bread is jointly influenced by starch crystallization, water distribution, protein conformation, enzyme activity, and fermentation characteristics.

## 4. Conclusions

This study systematically investigated the multi-scale structural changes in re-steamed bread with matcha under 0 °C cold chain conditions. It identified the optimal matcha concentration and clarified the trade-off between gluten protection and starch retrogradation inhibition. The 0 °C storage condition realistically simulates commercial cold chains, providing a practical reference for quality control of cold-staple foods.

Cold storage deteriorated re-steamed bread quality by increasing moisture mobility, decreasing gluten water-holding capacity, cleaving intermolecular disulfide bonds, destabilizing gluten secondary structure, and inducing starch retrogradation (rearrangement from disordered to ordered state). Adding matcha at ≤1% improved specific volume and reduced hardness by stabilizing disulfide bonds and protecting gluten. However, increasing matcha from 0.5% to 1.5% reduced specific volume, increased hardness, and promoted moisture migration. A substantial addition of phenolic compounds disrupted the gluten network and caused protein depolymerization. Matcha delays starch retrogradation in a dose-dependent manner.

## Figures and Tables

**Table 1:** Changes in the texture characteristics of re-steamed bread and short-range order of starch.

Matcha Concentration (%)	Time (d)	Hardness (g)	Springiness	Chewiness	Cohesiveness	Adhesiveness (g·s)	R_1045/1022_
0.0%	0	132.97 ± 8.84 ^dA^	0.75 ± 0.03 ^aB^	38.14 ± 2.74 ^dA^	0.40 ± 0.02 ^aC^	46.98 ± 2.82 ^aBC^	1.46 ± 0.02 ^cA^
	1	142.71 ± 4.53 ^dA^	0.64 ± 0.04 ^bC^	47.77 ± 2.90 ^cA^	0.39 ± 0.03 ^aB^	43.56 ± 2.47 ^abB^	1.54 ± 0.05 ^bA^
	3	159.06 ± 11.62 ^cA^	0.62 ± 0.05 ^bcB^	50.05 ± 3.06 ^cA^	0.38 ± 0.03 ^aA^	42.78 ± 3.02 ^abcB^	1.56 ± 0.02 ^abA^
	6	184.39 ± 9.79 ^bB^	0.60 ± 0.02 ^bcB^	62.78 ± 2.16 ^bA^	0.36 ± 0.02 ^abAB^	41.94 ± 1.73 ^bcC^	1.60 ± 0.05 ^abA^
	9	202.06 ± 8.34 ^aB^	0.56 ± 0.04 ^cB^	71.56 ± 1.90 ^aA^	0.33 ± 0.02 ^bA^	38.52 ± 1.67 ^cB^	1.63 ± 0.04 ^aA^
0.5%	0	126.46 ± 9.67 ^dA^	0.81 ± 0.01 ^aA^	33.36 ± 1.48 ^dB^	0.45 ± 0.03 ^aAB^	51.79 ± 3.22 ^aB^	1.42 ± 0.03 ^dAB^
	1	132.55 ± 8.05 ^cdA^	0.77 ± 0.03 ^aA^	34.32 ± 2.63 ^dC^	0.43 ± 0.02 ^abAB^	48.37 ± 2.53 ^abB^	1.51 ± 0.02 ^cAB^
	3	145.97 ± 6.35 ^bcA^	0.73 ± 0.02 ^bA^	46.62 ± 3.00 ^cA^	0.39 ± 0.03 ^bcA^	46.89 ± 3.10 ^bB^	1.55 ± 0.03 ^bcA^
	6	160.33 ± 7.62 ^abC^	0.64 ± 0.03 ^cAB^	55.29 ± 3.41 ^bB^	0.37 ± 0.02 ^cA^	46.22 ± 0.87 ^bB^	1.58 ± 0.02 ^abA^
	9	173.02 ± 11.63 ^aC^	0.59 ± 0.02 ^dAB^	67.75 ± 3.47 ^aA^	0.33 ± 0.02 ^dA^	45.88 ± 1.09 ^bA^	1.61 ± 0.03 ^aA^
1.0%	0	124.17 ± 10.57 ^cA^	0.82 ± 0.04 ^aA^	32.39 ± 0.67 ^eB^	0.48 ± 0.02 ^aA^	58.94 ± 3.32 ^aA^	1.38 ± 0.02 ^cBC^
	1	136.78 ± 9.58 ^bcA^	0.73 ± 0.05 ^bAB^	37.88 ± 2.16 ^dBC^	0.46 ± 0.01 ^aA^	57.75 ± 3.23 ^abA^	1.46 ± 0.06 ^bAB^
	3	148.76 ± 3.59 ^bA^	0.70 ± 0.02 ^bA^	47.23 ± 1.96 ^cA^	0.43 ± 0.01 ^bA^	56.71 ± 3.30 ^abA^	1.52 ± 0.06 ^abA^
	6	172.42 ± 4.93 ^aBC^	0.68 ± 0.02 ^bcA^	54.19 ± 3.26 ^bB^	0.39 ± 0.01 ^cA^	52.31 ± 3.38 ^bcA^	1.53 ± 0.04 ^abA^
	9	184.62 ± 12.99 ^aBC^	0.63 ± 0.04 ^cA^	68.32 ± 3.76 ^aA^	0.35 ± 0.01 ^dA^	46.94 ± 1.56 ^cA^	1.57 ± 0.01 ^aAB^
1.5%	0	133.73 ± 3.58 ^dA^	0.77 ± 0.02 ^aAB^	35.91 ± 2.53 ^dAB^	0.42 ± 0.02 ^aBC^	45.91 ± 1.83 ^aC^	1.35 ± 0.04 ^cC^
	1	141.09 ± 10.52 ^cdA^	0.70 ± 0.02 ^bBC^	41.49 ± 2.27 ^cB^	0.41 ± 0.03 ^aB^	43.44 ± 2.49 ^abB^	1.44 ± 0.04 ^bB^
	3	153.93 ± 10.54 ^cA^	0.68 ± 0.02 ^bA^	49.89 ± 2.84 ^bA^	0.39 ± 0.03 ^aA^	42.31 ± 3.25 ^abcB^	1.49 ± 0.05 ^abA^
	6	205.48 ± 8.28 ^bA^	0.63 ± 0.04 ^cAB^	68.50 ± 3.50 ^aA^	0.33 ± 0.03 ^bB^	39.85 ± 1.83 ^bcC^	1.52 ± 0.05 ^abA^
	9	225.86 ± 11.87 ^aA^	0.57 ± 0.03 ^dAB^	72.44 ± 2.61 ^aA^	0.30 ± 0.01 ^bB^	38.40 ± 1.44 ^cB^	1.54 ± 0.04 ^aB^

Values are means ± standard deviations. Letters represent a significant difference between the data in the same column (*p* < 0.05).

## Data Availability

The original contributions presented in the study are included in the article; further inquiries can be directed to the corresponding author.

## References

[1] Wang Y., Wang Y., Wang Z., Hao S., Xiang S., Su W., Tan M. (2025). Improvement of oat β-glucan on the quality deterioration of frozen wheat dough and steamed bread. Food Chem..

[2] Zhao B., Fu S., Li H., Li H., Liu C., Chen Z. (2022). Effect of storage conditions on the quality of frozen steamed bread. Int. J. Food Sci. Technol..

[3] Xu K., Chi C., She Z., Liu X., Zhang Y., Wang H., Zhang H. (2022). Understanding how starch constituent in frozen dough following freezing-thawing treatment affected quality of steamed bread. Food Chem..

[4] Wang P., Zou M., Gu Z., Yang R. (2018). Heat-induced polymerization behavior variation of frozen-stored gluten. Food Chem..

[5] Zhao B., Liu T., Hou L., Wu C., Fu S., Liu X., Li H., Liu K. (2024). Cryoprotective effect of curdlan on frozen wheat gluten: With respect to physicochemical properties and molecular structure. LWT-Food Sci. Technol..

[6] Zhao L., Li L., Liu G.-Q., Liu X.-X., Li B. (2012). Effect of frozen storage on molecular weight, size distribution and conformation of gluten by SAXS and SEC-MALLS. Molecules.

[7] Duan B., Guo J., Li P., Cheng B., Zhang G., Bai Z., Luo D., Cui C. (2024). Moisture, rheology and structure of deacetylated konjac glucomannan-treated dough and the performance of steamed bread under frozen storage. LWT-Food Sci. Technol..

[8] Tan J.-M., Li B., Han S.-Y., Wu H. (2023). Use of a compound modifier to retard the quality deterioration of frozen dough and its steamed bread. Food Res. Int..

[9] Liu Z., Chen J., Zheng B., Lu Q., Chen L. (2020). Effects of matcha and its active components on the structure and rheological properties of gluten. LWT-Food Sci. Technol..

[10] Yu K., Huang X., He W., Ma X., Wu D., Ding Z., Li P., Du C. (2023). Evaluation of the effects of thermal processing on antioxidant activity and digestibility of green tea noodles: Based on polyphenol stability and starch structure. J. Cereal Sci..

[11] Sivam A.S., Sun-Waterhouse D., Perera C.O., Waterhouse G.I.N. (2013). Application of FT-IR and Raman spectroscopy for the study of biopolymers in breads fortified with fibre and polyphenols. Food Res. Int..

[12] Lv Y., Li M., Pan J., Zhang S., Jiang Y., Liu J., Zhu Y., Zhang H. (2020). Interactions between tea products and wheat starch during retrogradation. Food Biosci..

[13] Xiao H., Lin Q., Liu G.-Q., Yu F. (2012). Evaluation of black tea polyphenol extract against the retrogradation of starches from various plant sources. Molecules.

[14] Zhao J.-W., Jie C., Hu W.-X., Ling C., Chen F.-S. (2022). Effect of polyphenolic compounds on starch retrogradation and in vitro starch digestibility of rice cakes under different storage temperatures. Food Biophys..

[15] Liu Y., He Y., Li L., Zhou Q., Du Q., Zhang H. (2025). Mechanism of structural and functional changes of matcha bread dough during freezing storage. Food Chem..

[16] Huang N., Ruan L., Zhang J., Wang Y., Shen Q., Deng Y., Liu Y. (2024). Improved physicochemical and functional properties of dietary fiber from matcha fermented by Trichoderma viride. Food Chem..

[17] Koláčková T., Sumczynski D., Zálešáková L., Šenkárová L., Orsavová J., Lanczová N. (2020). Free and bound amino acids, minerals and trace elements in matcha (*Camellia sinensis* L.): A nutritional evaluation. J. Food Compos. Anal..

[18] Shu Y., Yang R., Wen H., Cai X., Zhou L., Chen Y., Zhu Y., Xiang Y., Wu H. (2025). Total flavonoid of vine tea reduces neutrophil extracellular traps release by inhibiting PI3K-AKT-mTOR signaling pathway to treat ulcerative colitis. J. Funct. Foods.

[19] Jia Y., Cheng J.H., Tang X., Yu X., Sun D.W. (2026). Effects of hot air pretreatment on digestibility and quality of steamed bread. Food Chem..

[20] Chang X., Huang X., Tian X., Wang C., Aheto J.H., Ernest B., Yi R. (2020). Dynamic characteristics of dough during the fermentation process of Chinese steamed bread. Food Chem..

[21] Zhu X., Yuan P., Zhang T., Wang Z., Cai D., Chen X., Shen Y., Xu J., Song C., Goff D. (2022). Effect of carboxymethyl chitosan on the storage stability of frozen dough: State of water, protein structures and quality attributes. Food Res. Int..

[22] Ning J., Hou G.G., Sun J., Wan X., Dubat A. (2017). Effect of green tea powder on the quality attributes and antioxidant activity of whole-wheat flour pan bread. LWT-Food Sci. Technol..

[23] Kim T.H., Kwon C.W. (2026). Effect of anti-mold organic compounds on shelf-life extension and quality of yeast-fermented bread. LWT-Food Sci. Technol..

[24] Mei Z., Wang W., Feng X., Liu M., Peng S., Chen L., Chen H., Lin S. (2024). Effect of soluble oat β-glucan and tea polyphenols on the rheological properties and microstructure of wheat dough. LWT-Food Sci. Technol..

[25] Omedi J.O., Huang W., Zhang B., Li Z., Zheng J. (2019). Advances in present-day frozen dough technology and its improver and novel biotech ingredients development trends—A review. Cereal Chem..

[26] Zhu Y., Sang S., Fitriyanti M., Luo X., Khan I.M., Xu X. (2026). Application of seaweed polysaccharides in pre-baked frozen bread and their effect on frozen gluten. Food Struct..

[27] Zhu X., Chen Y., Zhang N., Luo Y., Peng R., Chen L., Xu J., Teng Y., Li B., Ding W. (2024). Chickpea peptide as a plant-based cryoprotectant in frozen dough: Insight into the water states, gluten structures, and storage stabilities. LWT-Food Sci. Technol..

[28] Wang C., Su G.Q., Wang X., Nie S.D. (2019). Rapid assessment of deep frying oil quality as well as water and fat contents in French fries by Low-Field Nuclear Magnetic Resonance. J. Agric. Food Chem..

[29] Qian X., Gu Y., Sun B., Wang X. (2021). Changes of aggregation and structural properties of heat-denatured gluten proteins in fast-frozen steamed bread during frozen storage. Food Chem..

[30] Wang C.C., Sheng Z., Zhang Y.H., Li Q., Jin P., Xie D.C., Min W.H., Zhang H.H. (2023). Effects of epigallocatechin-3-gallate on the structural hierarchy of the gluten network in dough. Food Hydrocoll..

[31] Yang S., Zhao X., Liu T., Cai Y., Deng X., Zhao M., Zhao Q. (2024). Effects of apple fiber on the physicochemical properties and baking quality of frozen dough during frozen storage. Food Chem..

[32] Xu M., Wu Y., Hou G.G., Du X. (2019). Evaluation of different tea extracts on dough, textural, and functional properties of dry Chinese white salted noodle. LWT-Food Sci. Technol..

[33] Guan E., Zhang T., Wu K., Yang Y., Bian K. (2023). Physicochemical properties and gluten structures of frozen steamed bread dough under freeze-thaw treatment affected by gamma-polyglutamic acid. Food Hydrocoll..

[34] Nawrocka A., Ruminska W., Szymanska-Chargot M., Niewiadomski Z., Mis A. (2022). Effect of fluorescence dyes on wet gluten structure studied with fluorescence and FT-Raman spectroscopies. Food Hydrocoll..

[35] Yu D., Zhang X., Zou W., Tang H., Yang F., Wang L., Elfalleh W. (2021). Raman spectroscopy analysis of the effect of electrolysis treatment on the structure of soy protein isolate. J. Food Meas. Charact..

[36] Lu H., Tian Y., Ma R. (2023). Assessment of order of helical structures of retrograded starch by Raman spectroscopy. Food Hydrocoll..

[37] Wang S., Li C., Copeland L., Niu Q., Wang S. (2015). Starch retrogradation: A comprehensive review. Compr. Rev. Food Sci. Food Saf..

[38] Jie B., Leshan Z., Xin J., Qiuyu Y., Jiawen P., Qi S., Junlin G., Xuebo L., Xiang D. (2024). Multi-scale structural changes and mechanistic analysis of wheat starch gels with common proteins in short-term retrogradation at low temperature. Food Hydrocoll..

[39] Shi J., Feng J., Wu Y., Zhang H., Li Z., Wu Z., Shen C., Yang H. (2026). Inhibition of starch retrogradation in fermented rice cakes during simulated cold chain logistics. Food Chem..

[40] Geng D.H., Tang N., Zhang N., Wang K., Asiamah E., Cheng Y., Zhao X. (2026). Effect of starch chain structures on the order, strength, and digestibility of retrograded starch structures: A review. Carbohydr. Polym..

[41] Yang Z., Li J., Ji Z., Sang S., Xu X. (2024). Effects of wheat starch content on its flour and frozen dough bread. Food Chem. X.

[42] Bordenave N., Hamaker B.R., Ferruzzi M.G. (2014). Nature and consequences of non-covalent interactions between flavonoids and macronutrients in foods. Food Funct..

[43] Du J., Yao F., Zhang M., Khalifa I., Li K., Li C. (2019). Effect of persimmon tannin on the physicochemical properties of maize starch with different amylose/amylopectin ratios. Int. J. Biol. Macromol..

[44] Zou Z., Chen X., Gao Y., Theppawong A., Liu Y., Sangsawad P., Bunyameen N., Deng S., Kraithong S., Gao J. (2025). Recent insights into functional, structural, and digestibility modifications of starch through complexation with polyphenols: A review. Food Chem..

[45] Yun L., Jianhui X., Jin T., Lili Y., Liya N. (2021). Matcha-fortified rice noodles: Characteristics of in vitro starch digestibility, antioxidant and eating quality. LWT-Food Sci. Technol..

[46] Lan Z., Haocun K., Bimal C., Xiaofeng B., Zhengbiao G., Yan H., Li C., Zhaofeng L., Caiming L. (2024). The substitution sites of hydroxyl and galloyl groups determine the inhibitory activity of human pancreatic α-amylase in twelve tea polyphenol monomers. Int. J. Biol. Macromol..

[47] He Q., Lv Y., Yao K. (2006). Effects of tea polyphenols on the activities of α-amylase, pepsin, trypsin and lipase. Food Chem..

[48] Nyambe-Silavwe H., Villa-Rodriguez J.A., Ifie I., Holmes M., Aydin E., Jensen J.M., Williamson G. (2015). Inhibition of human α-amylase by dietary polyphenols. J. Funct. Foods.

[49] Bojňanská T., Kolesárová A., Čech M., Tančinová D., Urminská D. (2024). Extracts with Nutritional Potential and Their Influence on the Rheological Properties of Dough and Quality Parameters of Bread. Foods.

